# Upregulated mRNA expression of desaturase and elongase, two enzymes involved in highly unsaturated fatty acids biosynthesis pathways during follicle maturation in zebrafish

**DOI:** 10.1186/1477-7827-6-56

**Published:** 2008-11-24

**Authors:** Sairatul D Ishak, Sze-Huey Tan, Hou-Keat Khong, Annette Jaya-Ram, Yee-Ling Enyu, Meng-Kiat Kuah, Alexander Chong Shu-Chien

**Affiliations:** 1 School of Biological Sciences, Universiti Sains Malaysia, 11800 Minden, Penang, Malaysia; 2 Malaysian Institute of Pharmaceuticals and Nutraceuticals, Level 1, J05 Building Science Complex, Universiti Sains Malaysia, 11800 Minden, Penang, Malaysia

## Abstract

**Background:**

Although unsaturated fatty acids such as eicosapentaenoic acid (EPA, C20:5n-3), docosahexaenoic acid (DHA, C22:6n-3) and arachidonic acid (ARA, C20:4n-6), collectively known as the highly unsaturated fatty acids (HUFA), play pivotal roles in vertebrate reproduction, very little is known about their synthesis in the ovary. The zebrafish (Danio rerio) display capability to synthesize all three HUFA via pathways involving desaturation and elongation of two precursors, the linoleic acid (LA, C18:2n-6) and linolenic acid (LNA, C18:3n-3). As a prerequisite to gain full understanding on the importance and regulation of ovarian HUFA synthesis, we described here the mRNA expression pattern of two enzymes; desaturase (fadsd6) and elongase (elovl5), involved in HUFA biosynthesis pathway, in different zebrafish ovarian follicle stages. Concurrently, the fatty acid profile of each follicle stage was also analyzed.

**Methods:**

mRNA levels of fadsd6 and elovl5 in different ovarian follicle stages were determined by semi-quantitative reverse transcription-polymerase chain reaction (RT-PCR) assays. For analysis of the ovarian follicular fatty acid composition, gas chromatography was used.

**Results:**

Our results have shown that desaturase displayed significant upregulation in expression during the oocyte maturation stage. Expression of elongase was significantly highest in pre-vitellogenic follicles, followed by maturation stage. Fatty acid composition analysis of different ovarian follicle stages also showed that ARA level was significantly highest in pre-vitellogenic and matured follicles. DHA level was highest in both late vitellogenic and maturation stage.

**Conclusion:**

Collectively, our findings seem to suggest the existence of a HUFA synthesis system, which could be responsible for the synthesis of HUFA to promote oocyte maturation and possibly ovulation processes. The many advantages of zebrafish as model system to understand folliculogenesis will be useful platform to further elucidate the regulatory and mechanism aspects of ovarian HUFA synthesis.

## Background

Polyunsaturated fatty acids (PUFA) have long been implicated in various aspects of vertebrate reproduction. Both the *n*-6 and *n-*3 PUFA influence reproductive processes through a variety of mechanisms, which includes provision of precursors for prostaglandin synthesis, inducement of steroidogenesis and regulation of transcription factors involved in reproductive process [1]. Among the PUFA, eicosapentaenoic acid (EPA, C20:5*n*-3), docosahexaenoic acid (DHA, C22:6*n*-3) and arachidonic acid (ARA, C20:4*n*-6), also collectively known as highly unsaturated fatty acids (HUFA) have been shown to play pivotal role in regulation of oocyte maturation and ovulation [2,3]. In aquaculture, supplement of HUFA in broodstock diet is essential to increase probability of spawning success [4,5]. *In vitro *studies using teleost follicles have reported the stimulation of maturation and ovulation by ARA [6,7]. Besides the provision of adequate dietary ARA, EPA and DHA levels respectively, studies have also demonstrated the importance of having a balanced dietary ratio of these three HUFA for better reproductive performance [4,5]. Studies have shown that several marine fish species selectively transfer HUFA from muscle reserves to oocytes as preparation of long spawning season [8,9]. In addition, comparative analysis of fatty acid composition in ovary of wild and captive farmed fish have proposed inferior or imbalanced ratio of HUFA as the main reason for poor spawning performance of farmed broodstock [3,10].

Freshwater fish species possess the capacity to synthesize EPA and DHA from linolenic acid (LNA, 18:3*n*-3) and ARA from linoleic acid (LA, 18:2*n*-6) respectively, through two separate pathways involving desaturation and elongation of their respective precursors [11,12]. The extent to which animals, including fish, can convert LNA and LA, to HUFA differs according to species and depend on the activities of the desaturase and elongase enzymes. In most freshwater fish, EPA is synthesized from LNA by desaturation at the Δ6 position, followed by a 2-carbon elongation, and a further desaturation at the Δ5 position. Subsequently, synthesis of DHA from EPA is believed to proceed via a C24 intermediate, requiring two successive elongations to 22:5*n*-3 and 24:5*n*-3, followed by desaturation at the Δ6 position, and lastly a chain shortening process to produce DHA [13]. Production of ARA involves desaturation at Δ6 position of LA followed by a 2-carbon elongation process and lastly a desaturation at the Δ5 position [13]. We have previously reported the presence of desaturase and elongase mRNA in oocytes of two freshwater fish species, swordtail and zebrafish [14,15]. Despite the known importance of PUFA in vertebrate reproduction and the proven selective accumulation of unsaturated fatty acids in oocyte and fertilized eggs, very little is known about the existence, role and regulation of the HUFA biosynthesis system in ovary [16].

In addition to its value as a model organism in developmental biology, the zebrafish is emerging as a useful model to understand oogenesis and folliculogenesis [17]. The female zebrafish is a prolific spawner with the oocytes easily differentiated to 4–5 distinct follicle stages. In addition, gonadotropin and steroid induced *in vitro *maturation of zebrafish oocytes have also been developed [18,19]. Accordingly, transcriptome and proteome analysis have been carried out to map vital molecular pathways responsible for both maturation and ovulation processes in zebrafish [20,21]. Based on the importance of HUFA in reproduction, we reason that localized desaturation and elongation activities in oocyte could potentially be a source of HUFA for maturation and ovulation processes. As a prerequisite to this hypothesis, we investigated the mRNA levels of desaturase and elongase and unsaturated fatty acids composition in different zebrafish follicle stages. Collectively, the mRNA expression pattern and fatty acid composition indicate the synthesis of ARA and DHA in zebrafish follicles as they enter into maturation and ovulation stages.

## Methods

### Fish maintenance

Adult female zebrafish (*Danio rerio*) aged 4 months old were maintained in Aquaculture Research Complex, Universiti Sains Malaysia. Fish were fed till satiation twice daily with a combination of *Artemia *nauplii and frozen bloodworms. Breeding was carried out every 3 days in spawning tanks according to Westerfield [22]. Male zebrafish were kept in separate tanks and fed frozen bloodworms twice a day and selected randomly for spawning.

### Sampling of ovarian follicles

Ovaries from 10–15 female zebrafish were removed after fish was anesthetized and humanely sacrificed, and subsequently placed in 60% Leibovitz L-15 medium. The follicles of different stages were then manually isolated using fine-tipped forceps and grouped into following stages according to classifications by Selman *et al*. [23]: pre-vitellogenic (PV; 0.14–0.34 mm), early and mid-vitellogenic (EV; 0.34–0.61 mm), fully-grown but immature (LV; 0.62–0.70 mm), mature (M; ~0.70 mm, follicles become translucent following *in vivo *germinal vesicle breakdown) and ovulated oocytes (O; ovulated into ovarian lumen). The PV, EV and LV stages were sampled on same day, while matured follicles were obtained the following morning before daylight; and ovulated oocytes during the first hour of daylight by stripping of females.

### Lipid extraction and fatty acid analysis

Fatty acid determination was carried out on staged ovarian follicles as described above. Total lipid was extracted from samples by homogenization in chloroform/methanol (2:1, v/v) methylated and transesterified with boron trifluoride in methanol [24]. Fatty acid methyl esters (FAME) were separated and quantified by gas chromatography (Automatic System XL; Perkin Elmer, USA) equipped with a flame ionization detector and a 30 m × 0.25 mm fused silica capillary column (Omegawax 250; Supelco, USA). Helium was used as carrier gas and temperature programming was from 50 to 220°C at 4°C/min, and then held at 220°C for 35 min. The injector and detector temperatures were set at 250°C and 260°C, respectively. Individual methyl esters were identified by comparison to known standards and by reference to published data [25]. Composition of LNA, LA, ARA, EPA and DHA was then calculated as percentage of total fatty acid for each staged ovarian follicles.

### RNA extraction and semi-quantitative RT-PCR analysis

Total RNA was extracted from approximately 100 mg of each ovarian follicle stage using TRI Reagent^® ^(Molecular Research Center, USA) according to the manufacturer's specifications. The total RNA concentration was determined by A260/A280 measurement and subsequently pretreated with RQ1 RNase-free DNase (Promega, USA). Two micrograms of the DNase treated-total RNA was reverse transcribed into first-strand cDNA at 42°C for 1 h in a total volume of 20 μl containing 1× M-MLV RT Reaction Buffer, 25 ng of Random Primers, 0.5 mM of each dNTP, 24 U of Recombinant RNasin^® ^Ribonuclease Inhibitor and 200 U of M-MLV Reverse Transcriptase (Promega, USA). For PCR, 2 μl of first-strand cDNA was subjected to a reaction mixture with total volume of 25 μl consisting 1× Green GoTaq^® ^Flexi Buffer, 2.0 mM MgCl_2_, 0.2 mM each dNTP, 0.4 μM of each primers and 1 U of GoTaq^® ^DNA Polymerase (Promega, USA). The PCR program used was an initial denaturation at 94°C for 1 min; amplification cycles of 94°C for 30 s, 58°C for 30 s, 72°C for 30 s; with an extension at 72°C for 10 min. PCR products were visualized on agarose gel containing ethidium bromide. The product yield was quantified using GeneTools software (Syngene, USA).

Specific primers for RT-PCR amplification were designed according to published zebrafish desaturase, elongase, and histone H2A (as housekeeping gene) mRNA sequences. The primers were designed to span on different exons to eliminate the possibility of genomic contamination. The sequences of the primers and size of amplicons for each gene are presented in Table 1. The PCR products were sequenced, which revealed that elongase, desaturase and histone H2A fragments derived from RT-PCR analysis were virtually 100% identical to the published zebrafish putative delta-6 fatty acyl desaturase mRNA [*fadsd6*, GenBank: AF309556]; zebrafish ELOVL family member 5, elongation of long chain fatty acid mRNA [*elovl5*, Genbank: NM_200453] and zebrafish H2A histone family, member Z mRNA [*h2afz*, Genbank: XM_001921153] respectively, which further verified the specificity of the primers used in RT-PCR analysis. Semi-quantitative RT-PCR assays were validated by running series of PCR reactions to determine number of cycles which generates half-maximal PCR reaction.

**Table 1:** Oligonucleotide primers used in semi-quantitative RT-PCR analysis.

Gene	Amplicon	Sense 5'-3'	Anti-sense 5'-3'
*fadsd6*	336 nt	CATCACGCTAAACCCAACATC	GCTCTCCATAAACCTGACGAAA
*elovl5*	474 nt	TGGACACCTTCTTCTTCATCC	TCTTCTCGCTGGACATCACTC
*h2afz*	159 nt	AGTTTTGGAGTTGGCAGGAAAT	AGAGACTTGTGGATGTGTGGAAT

### Statistical analysis

Data are presented as mean ± SEM of three replicates. The significance of the differences between group of means were determined by one-way analysis of variance (ANOVA) at *P *< 0.05 and was then subjected to Tukey's HSD post hoc test (SPSS Inc., USA).

## Results

### Fatty acid composition analysis

**Figure 1:**
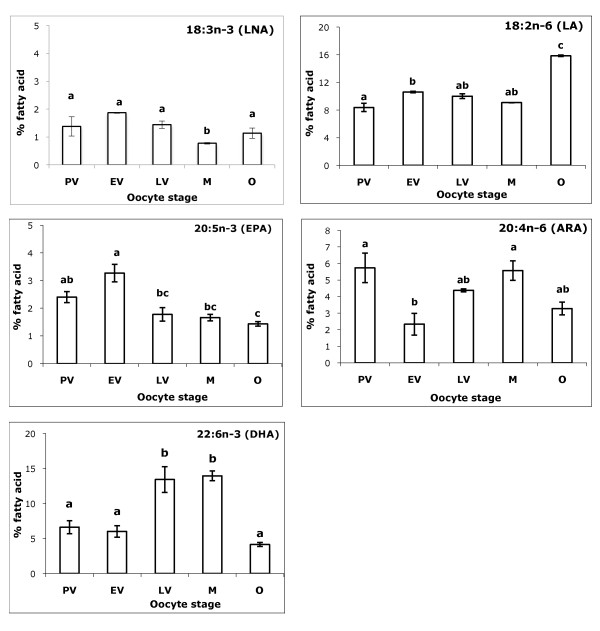
**Fatty acid profiles of HUFA (%) in five ovarian follicle stages**. PV represents pre-vitellogenic stage, EV represents early and mid-vitellogenic stage, LV represents fully-grown but immature stage, M represents matured oocyte stage and O represents ovulated oocyte stage. Each value represents mean ± SEM of triplicates at the significant level *P *< 0.05 using Tukey's HSD. Mean values with different alphabets are significantly different.

Figure 1 shows that ARA level was initially high in PV follicles, followed by a decrease during EV stage. As vitellogenesis progresses, ARA level raised and peaked at maturation stage. As for EPA, EV oocytes showed highest level of expression while latter oocyte stages showed a decreasing trend. The level of DHA was low during early vitellogenic stages, followed by a significant increase in the LV and matured follicles, before dropping again in the ovulation stages. Level of LNA was low in all follicle stages, while level of LA was significantly highest at the ovulation stage.

### Validation of semi-quantitative RT-PCR assays

**Figure 2:**
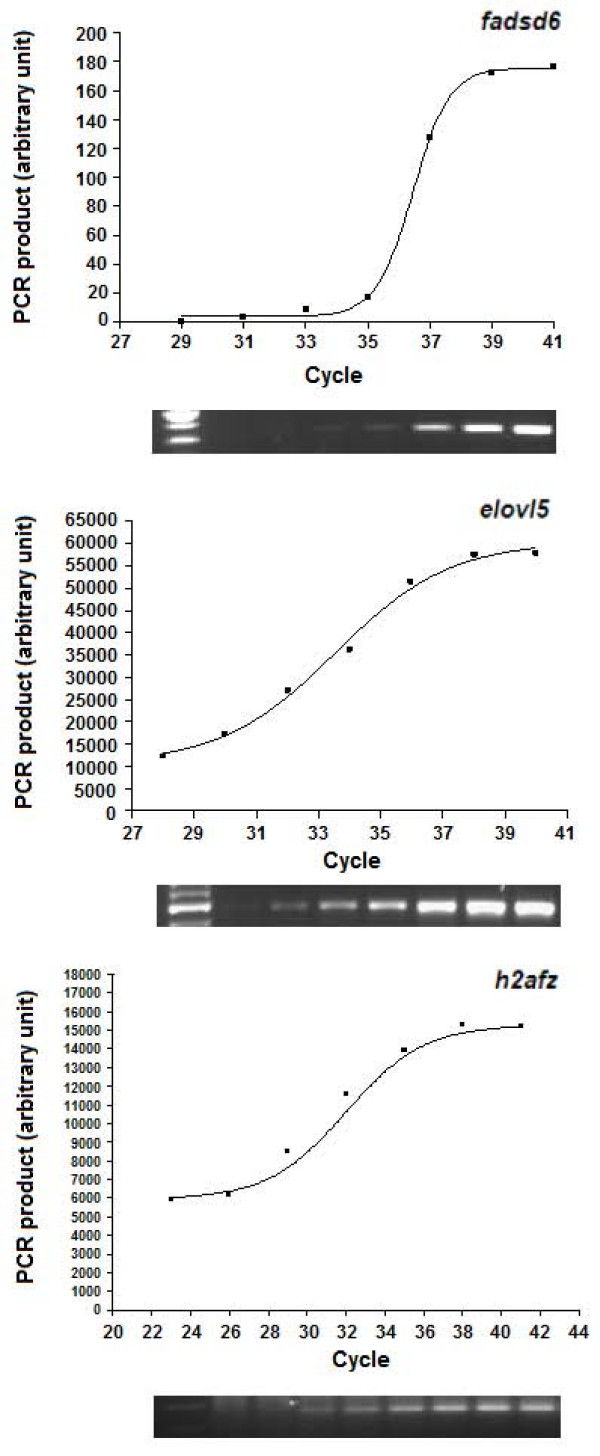
Validation of semi-quantitative RT-PCR assays for *fadsd6 *(desaturase), *elovl5 *(elongase) and *h2afz *(histone H2A) genes.

In order to quantify the gene expression accurately, series of RT-PCR reactions were carried out to ensure the reactions were within the exponential phase of amplification. Samples were collected at different cycles and analyzed by electrophoresis for *h2afz*, *fadsd6 *and *elovl5 *genes. Band intensities were analyzed using GeneTools software (Syngene, USA) and graphs were constructed using GraphPad Prism v3.0 (GraphPad Software Inc., USA). Based on the constructed graphs (figure 2), the half-maximal cycles are 32, 37 and 35 for *h2afz*, *fadsd6 *and *elovl5 *respectively.

### Semi-quantitative RT-PCR assays

**Figure 3:**
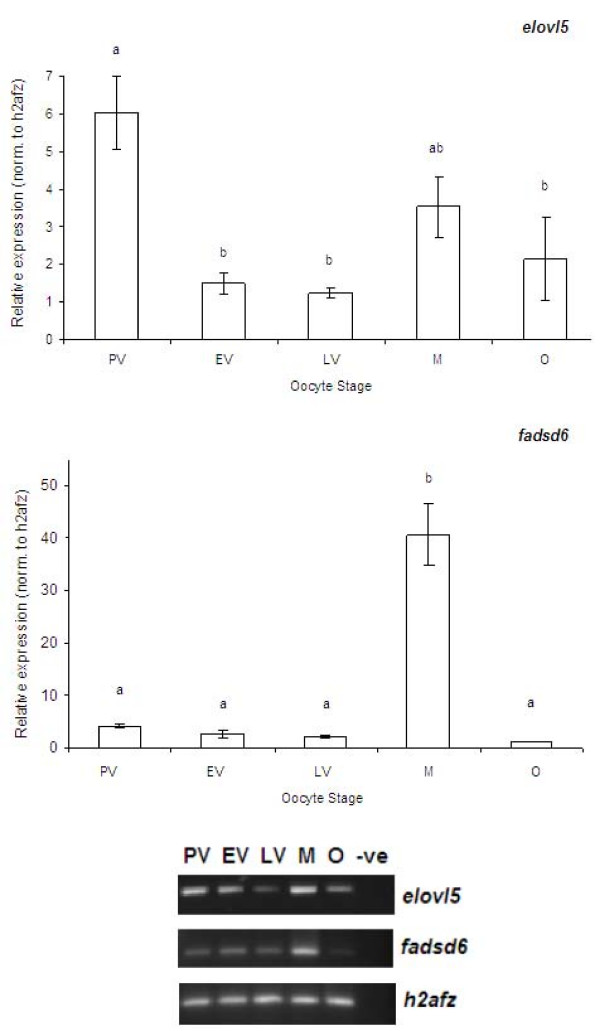
**Expression of *elovl5 *and *fadsd6 *normalized to *h2afz *during the zebrafish follicles development from PV to ovulation stage**. PV represents pre-vitellogenic stage, EV represents early and mid-vitellogenic stage, LV represents fully-grown but immature stage, M represents matured oocyte stage and O represents ovulated oocyte stage. – ve represents negative control, with sterile water as template for RT-PCR. Each value represents mean ± SEM of three PCR runs at the significant level *P *< 0.05 using Tukey's HSD. Mean values with different alphabets are significantly different. Representative of the electrophoretic images of RT-PCR is shown at the lower panel.

Zebrafish follicles were collected from 10–15 fishes for semi-quantitative RT-PCR analysis. Figure 3 shows relative expression and RT-PCR electrophoretic images of *elovl5 *and *fadsd6*. Values are expressed as arbitrary units of *elovl5 *and *fadsd6 *normalized against the expression levels of *h2afz *from the same template. For desaturase, significantly highest expression was obtained at maturation stage. Elongase expression was highest at pre-vitellogenic stage, followed by a significant decrease in subsequent vitellogenic stage follicle, before eventually increasing in expression in maturation stage.

## Discussion

Oocyte fatty acid composition is species-specific in terms of its apparent abundance and utilization. Among the fatty acids, EPA, DHA and ARA, have been implicated in many key aspects of reproduction such as precursors for prostaglandin synthesis, steroidogenesis and regulation of essential reproductive related transcription factors [1]. In vertebrates, depending on species, HUFA are either generated by *de novo *synthesis or are absorbed from the diet. Freshwater fish species possess the ability to synthesize EPA, DHA and ARA from their respective precursors via 2 separate pathways involving desaturation and elongation processes. In contrast, this ability is limited or non-present in marine species, which also meant that the supply of EPA, DHA and ARA in these species comes exclusively from dietary intake. Despite the known importance of unsaturated fatty acids in oocytes, very little is understood on the presence, role and regulation of their synthesis in oocytes. In contrast, this subject has been well investigated in the mammalian testis [26]. As a prerequisite to address this issue, we showed here the EPA, ARA and DHA composition of different zebrafish follicle stages and mRNA expression pattern of desaturase and elongase, 2 enzymes involved in the HUFA biosynthesis pathways.

In this present study, histone H2A was used as a housekeeping gene for validation of desaturase and elongase expression in different oocyte stages. Expression of commonly used housekeeping genes such as beta-actin, acidic ribosomal phosphoprotein, elongation factor-1 alpha and glyceraldehyde 3-phosphate dehydrogenase have been reported to show decreasing pattern as follicles matured, making them unsuitable for the traditional normalization method for gene expression determination [27,28]. A proposed solution to this decreasing pattern was to create an equivalent average for each follicular stage prior to normalization of gene of interest [27,29-31]. Recently histone H2A was also used as reference gene in a study on gene expression in trout oocyte stages [32]. Our results showed the consistency in expression of zebrafish histone H2A in the 5 follicle stages analyzed, confirming its suitability as a reference gene.

There was an increase in ARA and DHA levels in matured follicles. The elevated levels of ARA is in agreement with a study detailing effect of maturation process on lipid profiles of *Bufo arenarum *oocyte, which reported significant increase in ARA in phosphatidylserine[33]. ARA is the precursor for eicosanoids, a group of prostaglandins known to have pivotal roles in oocyte maturation and ovulation [34-36]. The finding showing fortification of matured zebrafish oocytes with ARA strongly indicate the capacity of oocytes to synthesize eicosanoids for follicular maturation and ovulation. In oocytes of pig, cattle and sheep, ARA, together with LA was reported to be the most abundant polyunsaturated fatty acid [7]. ARA has also been implicated to play essential role in steroidogenesis[37]. In addition, the trend of increasing ARA level coupled with decreasing EPA level in maturing zebrafish oocytes could be due to the need to increase the ratio of ARA:EPA as EPA may inhibit ARA-based prostaglandin synthesis due to increased competition between EPA with ARA for binding to prostaglandin H synthase [5,38]. In fish farming, low ARA:EPA ratio in ovaries and eggs have often been cited as a reason for poor performance in captive broodstock [3,10]. Studies in warm and cold water fish species have shown that DHA, together with EPA inhibit gonadotropin-stimulated steroidogenesis, implying the importance of these two HUFAs as regulator of maturation in fish ovary [39].

Semi-quantitative RT-PCR analysis of elongase gene in different zebrafish oocyte stages revealed high levels of mRNA during the initial PV stage, followed by a reduction in expression during the EV and LV stages. There was a slight increase in expression during maturation. To our knowledge, this is the first report on differential mRNA expression of a long chain fatty acid elongase in different oocyte stages. Although actual enzymatic activities of both genes were not measured here, studies on several fish species have shown that expression of desaturase and elongase mRNA correlates with their enzymatic activities [14,15,40]. The primers utilized for elongase amplification in this study yielded a partial cDNA sequence, which was 100% identical to a zebrafish *elovl *family member 5. Elsewhere, functional characterization of zebrafish *elovl5 *gene using recombinant *Saccharomyces cerevisiae *showed that this enzyme display broad substrate specificities, with ability to elongate monounsaturated fatty acids and the C18, C20 and C22 PUFA [41]. In addition, the zebrafish *elovl5 *is able to perform all the elongation steps necessary to produce DHA and ARA from LA and LNA respectively [41]. In zebrafish, *elovl5 *was also reported to have the capability to elongate the C20 and C22 fatty acids to tetracosapentaenoic acid (C24:5*n*-3). This is important as study in rat liver have shown that C24:5*n*-3 can be further processed to the physiologically important DHA [42]. Elsewhere, a mouse ovulatory-selective cDNA library reported increased expression of elongase, which indicates its potential role during ovulation processes [43]. Recently, it has been shown the mouse *elovl6 *is regulated by the sterol regulatory element binding proteins (SREBP) [44]. Elsewhere, SREBP have also been implicated in the development of avian follicles [45]. Similarly, the lipid x receptor (LXR), another regulator of elongase is also vital for oocyte maturation, as LXR-deficient mice showed reduced fertility and inability of oocytes to resume meiosis [46]. Therefore, a possible role for SREBP and LXR in follicle maturation could be regulation of elongase to synthesize essential unsaturated fatty acids, although further experiments will be required to confirm this.

There was also an increase in zebrafish putative delta-6 fatty acyl desaturase (*fadsd6*) mRNA levels in matured zebrafish follicles. This gene has been characterized and is reported to encode for a desaturase enzyme with bifunctional activities, capable of both Δ5 and Δ6 activities, with slight preference towards *n*-3 substrates and towards the Δ6 desaturation [47]. In rat, characterization of expression of stearoyl-coenzyme A desaturase, the rate limiting enzyme in the biosynthesis of monounsaturated fatty acids from saturated fatty acids seems to suggest the importance of this enzyme in production of unsaturated fatty acids to enable follicular maturation [48]. The decrease in expression of desaturase and elongase in ovulation stage could be due to a negative feedback loop from the high levels of EPA, DHA and ARA in the maturation stage. In human, high levels of PUFA have been shown to negatively regulate desaturase mRNA expression[49].

Although we do not rule out the possibility of more isoforms of fatty acid desaturase or elongase genes in zebrafish ovaries, it is conceivable that both the genes investigated here are capable of producing all three HUFA from the C18 fatty acids in ovary. Functional studies on both these genes have shown activities encompassing the whole pathway of production of EPA, DHA and ARA from LA and LNA respectively [41,47]. This is in contrast with other teleost species such as salmon, which possess specific desaturase enzymes having either only Δ5 or Δ6 activity respectively [50]. In addition, low desaturase and elongase activities have been cited as main reason for limited HUFA biosynthesis capabilities in marine species like cod and turbot [51,52]. Thus, it will be important to actually determine the origins of the HUFAs in oocyte, as these fatty acids are also transported from reserves in muscles as compared to those presumably synthesized in oocytes.

Future experiments will be required to further support the suggestion of localized HUFA biosynthesis activities within the oocytes. In addition to measurement of actual enzymatic activities of both desaturase and elongase, it is also possible to elucidate their function by using a morpholino knockdown technique to block the translation process of these two genes in the oocyte. This technique has been used to confirm the function of candidate genes essential for oocyte maturation in zebrafish follicles [53,54]. Since the zebrafish oocyte fatty acid composition can be manipulated by dietary fatty acid intake, it is possible to combine the morpholino method with different dietary fatty acid treatment, which will provide insight on the importance of desaturase and elongase under different condition of dietary fatty acid intake.

## Conclusion

In conclusion, we demonstrate here the differential expression of desaturase and elongase genes in different zebrafish follicular stages. Desaturase expression showed a significant increase during maturational stage, which also coincides with increase in ARA and DHA levels. Elongase expression was initially high in pre-vitellogenic follicles, decreased during vitellogenesis and subsequently increased during maturation, although not significantly. Overall, these findings suggest the possibility of a localized HUFA biosynthesis in zebrafish ovary, suggesting the need for further studies to detail the actual elongation and desaturation enzymatic activities, functional significance and regulatory aspects of these activities.

## Competing interests

The authors declare that they have no competing interests.

## Authors' contributions

SDI, SHT and HKK carried out the RNA extraction, PCR and data interpretation. AJR and YLE assisted in separation and identification of oocyte stages. Design of primers was done by MKK. AC conceived the study, designed the experiments and structured the manuscript. All authors proofread and approved the final submitted manuscript.
